# Recovery of Valuable Solutes from Organic Solvent/Water Mixtures via Direct Contact Membrane Distillation (DCMD) as a Non-Heated Process

**DOI:** 10.3390/membranes11080559

**Published:** 2021-07-23

**Authors:** Yuki Suga, Ryosuke Takagi, Hideto Matsuyama

**Affiliations:** 1 Research Center for Membrane and Film Technology, Department of Chemical Science and Engineering, Kobe University, Nada, Kobe 657-8501, Japan; suga.yg@om.asahi-kasei.co.jp (Y.S.); takagi@harbor.kobe-u.ac.jp (R.T.); 2 Asahi Kasei Corporation, Chiyoda-ku, Tokyo 100-0006, Japan

**Keywords:** membrane distillation (MD), direct contact MD, hollow fiber membrane, polyvinylidene difluoride, organic solvent, ethanol, acetonitrile

## Abstract

Recently, the demand for the recovery of valuable solutes from organic solvents/water mixtures has increased in various fields. Furthermore, due to the abundance of heat-sensitive valuable solutes, the demand for non-heated concentration technologies has increased. In this study, the direct contact membrane distillation (DCMD) using hydrophobic polyvinylidene difluoride (PVDF) hollow fiber membranes was investigated to confirm the possibility of recovering valuable solutes from organic solvents/water mixtures as a non-heated process. The DCMD with 1000 ppm NaCl aqueous solution achieved 0.8 kg/m^2^·h of vapor flux and >99.9% of NaCl retention, even at feed and coolant temperatures of 25 and 10 °C, respectively. Furthermore, when DCMD was conducted under various conditions, including feed temperatures of 25, 35 and 45 °C, and organic solvent concentration of 15, 30 and 50 wt%, using ethanol/water and acetonitrile/water mixtures containing 1000 ppm NaCl. A surfactant was also used as a valuable solute, in addition to NaCl. As a result, it was found that the total vapor flux increased with increasing temperature and concentration of organic solvents, as the partial vapor pressure of the organic solvents increased. Additionally, no solute leaked under any condition, even when the surfactant was used as a valuable solute.

## 1. Introduction

Recently, the demand for the recovery of valuable solutes from organic solvents/water mixtures have increased in numerous areas, including chemical and pharmaceutical production [1,2]. For instance, organic solvents/water mixtures are used in the synthesis and purification processes of peptides [3]. Since many of these peptides are heat-sensitive, the demand for non-heated concentration technology has increased.

Numerous studies on organic solvent nanofiltration (OSN) as a non-heated concentration technology have been reported [4,5]. OSN is regarded as an energy-efficient concentration method due to the absence of phase transition [6]. Additionally, since OSN is a membrane technology, it is easier to scale up than the conventional distillation technologies [1]. However, because OSN membranes separate solutes by size, it is difficult to concentrate valuable resources that are smaller than the membrane pore size without incurring losses, since they easily pass through the OSN membranes [7,8,9].

Membrane distillation (MD) is one of the distillation technologies, in which water vapor and the vapor of organic solvents are permeated through a membrane using a vapor pressure difference across the membrane as the driving force [10]. Theoretically, MD can separate any solutes from a solvent, as long as the solutes are non-volatile. Thus, MD can separate small solutes which cannot be separated using OSN. In addition, it is possible to concentrate the feed at temperatures below ambient temperature, if the vapor pressure difference between the feed and permeate sides of the membrane is sufficient. Additionally, MD shares many of the same advantages as other membrane technologies, including a simpler system and greater scalability than conventional distillation technologies.

Almost all MD operating conditions reported in previous studies report a feed temperature higher than 40 °C and a feed solution that did not contain any organic solvents [11]. However, in applications such as chemical and pharmaceutical manufacturing processes, the operating temperature of the MD must be below ambient temperature to avoid the deterioration of valuable resources due to heat. In addition, organic solvents are frequently present in aqueous solutions. Thus, there are two challenges associated with applying MD technology to the process of chemical and pharmaceutical recovery. One issue is low vapor flux through the membrane as a result of a small vapor pressure difference caused by the low feed temperature. The other issue is membrane wetting caused by organic solvents. When the membrane is wet, liquids permeate through the membrane, resulting in the leak of solutes [12].

The vapor flux of MD, J (kg/m^2^·h) is proportional to the vapor pressure difference between the feed side and the permeation side and is given by Equation (1) [13,14].

(1) $$J=\alpha \left(P_{\mathrm{feed}}-P_{\mathrm{permeate}}\right)$$

Here, α (kg/m^2^·h · kPa) is the vapor permeation coefficient. $P_{\mathrm{feed}}$ (kPa) and $P_{\mathrm{permeate}}$ (kPa) are the saturated vapor pressures of the feed side and of the permeation side, respectively. ($P_{\mathrm{feed}}$ − $P_{\mathrm{permeate}}$) should be positive, since this is the driving force of vapor permeation. Equation (1) suggests that as the temperature of the feed decreases, it becomes more difficult to acquire enough vapor pressure difference, as the saturated vapor pressure decreases. Therefore, the vapor flux of MD will become extremely low under low feed temperature conditions. Only a few studies of MD operation at low feed temperatures have been reported so far [14]. Furthermore, even if they were successful in MD operation, the vapor flux was extremely low. For example, Macedonio et al. performed direct contact MD (DCMD) operation at 30 °C and 25 °C for the feed and permeate, respectively [13]. Additionally, they used the commercial polypropylene flat sheet membrane and obtained 0.2 kg/m^2^∙h as the water vapor flux.

Membrane wetting is another severe problem in MD operation. Membrane wetting occurs when a transmembrane pressure becomes higher than the liquid entry pressure (LEP). LEP (MPa) is the pressure required for the liquid to penetrate into the membrane pore [15]. LEP is given by Equation (2).

(2) $$\mathrm{LEP}=\frac{-2B\sigma_{L}cos\theta }{r_{max}}$$

Here, B is a geometric factor determined by pore structure, for example, B = 1 for cylindrical pores. $\sigma_{L}$ is surface tension of a liquid, θ a contact angle and $r_{max}$ a maximum pore radius of membrane. In general, organic solvents decrease the surface tension of liquid in comparison with water, subsequently decreasing LEP [14]. Thus, MD is difficult to be applied for recovering valuable solutes from organic solvent/water mixtures, since the membrane is easily wetted.

So far, few studies have been conducted that report the use of MD against organic solvent/water mixtures. In a few instances when an organic solvent/water mixture is used, the flux is extremely low as a result of improved LEP to avoid wetting. For example, Banat et al. performed an air gap MD (AGMD) operation using a polyvinylidene difluoride (PVDF) flat sheet membrane and treated an ethanol aqueous solution [16]. They obtain approximately 1 kg/m^2^∙h flux using 42 °C of 3.3 wt% EtOH aqueous solution as the feed. Additionally, Gupta et al. performed sweep gas MD (SGMD) using a composite membrane containing carbon nanotube to concentrate the iso-propanol aqueous solution [17].

The current study attempts to improve vapor flux from two perspectives: the membrane and the MD operation method. Regarding MD membrane, a hydrophobized PVDF hollow fiber (HF) membrane with a high vapor flux and high LEP, fabricated in our previous study, was used as the MD membrane [18]. It is expected that by using this membrane, the vapor will permeate efficiently even at low temperatures where the vapor pressure difference is low. Additionally, the membrane will scarcely become wet with the feed, which contains organic solvents, because the membrane will maintain sufficient LEP by the combination of high hydrophobicity and small maximum pore size, even if the surface tension of the feed aqueous solution becomes low due to the contained organic solvents.

**Figure 1:**
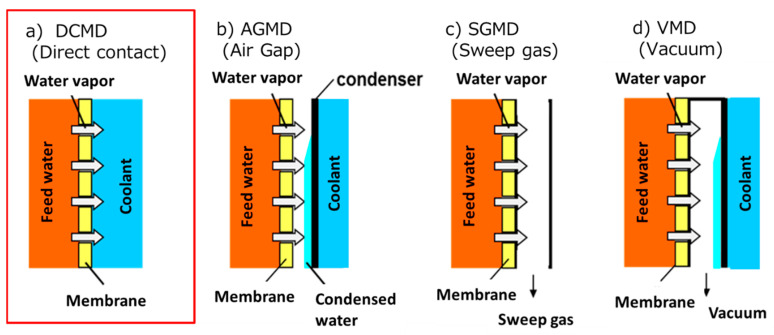
Schematics of typical membrane distillation (MD) operation setups. (**a**) Direct contact MD (DCMD), (**b**) Air gap MD (AGMD), (**c**) Sweep gas MD (SGMD), (**d**) Vacuum MD (VMD). DCMD was used in this paper.

Regarding MD operation, it is important to choose the method that allows for a large vapor pressure difference across the membrane while reducing the transmembrane pressure simultaneously. MD is classified into several types based on the method of operation. Figure 1 shows the schematics of typical MD setups [19]. A direct contact MD (DCMD) (Figure 1a) is the simplest MD operation method and has been used in numerous earlier studies [10]. In DCMD, a feed water contacts with a coolant through a membrane and the pass where vapors permeate through is the shortest. Thus, the vapor flux of DCMD becomes very high. However, the heat efficiency is low because heat conduction is most likely to occur through the membrane, and temperature polarization reduces the flux [20]. By creating an air gap between the membrane and the cooling section (Figure 1b), an air gap MD (AGMD) suppresses heat conduction through the membrane [21]. Thus, the heat efficiency is higher than DCMD. However, the vapor flux is lower than that of DCMD, because of a lower vapor pressure difference. To speed up a diffusion transfer of vapor, a sweep gas MD (SGMD) (Figure 1c) and a vacuum MD (VMD) (Figure 1d) are designed [22,23]. In SGMD, a sweep gas is flowed through the air gap part, while the air gap part is decompressed in VMD. Thus, by using SGMD or VMD, it is possible to achieve both high vapor flux and low heat conduction. However, VMD requires a high vacuum, and SGMD requires a large amount of dry air to be supplied.

As discussed above, while each process has merits and demerits, DCMD is chosen to recover valuable solutes from an organic solvent/water mixture because the high vapor flux can be obtained simply by flowing water that is cooler than the feed. In terms of membrane wetting, DCMD has a lower transmembrane pressure than other MD processes. Therefore, there is a high possibility that it can be operated even if the LEP decreases due to the organic solvent contained in the feed.

## 2. Materials and Methods

### 2.1. Materials

Solef 6010 (SOLVAY, Brussels, Belgium) was used as the PVDF resin [18,24]. AEROSIL-R972 (NIPPON AEROSIL, Tokyo, Japan) was the hydrophobic silica, and functioned as a pore-forming agent. The PVDF polymer was diluted using Di (2-ethylhexyl) phthalate (DOP) and dibutyl phthalate (DBP). Following fabrication, CH_2_Cl_2_, EtOH and NaOH were used to wash the membrane. The membrane porosity was determined using 1-Butanol., NaCl was used as a model electrolyte in the feed solution. Sodium dodecyl sulfate (SDS) was used as a model surface-active solute. All of these chemicals were purchased from FUJIFILM Wako Pure Chemical Corporation, Osaka, Japan. The fluoropolymer FS-392B (Fluoro Technology Co. Ltd., Aichi, Japan), was used as the hydrophobic agent [25,26].

### 2.2. Fabrication of Hydrophobized PVDF Membrane

#### 2.2.1. Fabrication of PVDF Hollow Fiber Membrane

The PVDF hollow fiber (HF) membrane was fabricated using the thermally induced phase separation (TIPS) method and was subsequently treated with a hydrophobic agent to produce PVDF HF with a high LEP [18]. At first, the PVDF HF membrane was fabricated using the TIPS method described in the patent [18,24]. The dope solution was comprised of hydrophobic silica, DOP, DBP and PVDF at a weight ratio of 23:31:6:40. This was melted at 240 °C and extruded through the outer slit of a double-orifice spinneret. Simultaneously, nitrogen gas was discharged from the inner slit of the spinneret as a hollow part formation fluid. The extruded dope was then introduced into a water bath (40 °C) through a 20 cm air gap and wound up at a rate of 20 m/min. Following that, the membrane was immersed in CH_2_Cl_2_ to remove DOP and DBP, and then dried. Subsequently, the membrane was immersed in a 50 wt% EtOH aqueous solution, and 5 wt% NaOH aqueous solution for 1 h at 40 °C to remove silica. The analysis of the membrane composition revealed that silica particles were completely removed. The PVDF HF membrane was finally obtained after washing with water and drying. After inserting PVDF HF membranes into a lab-scale module, they were treated with a hydrophobic agent as described in Section 2.2.3.

#### 2.2.2. Preparation of Membrane Modules

**Figure 2:**
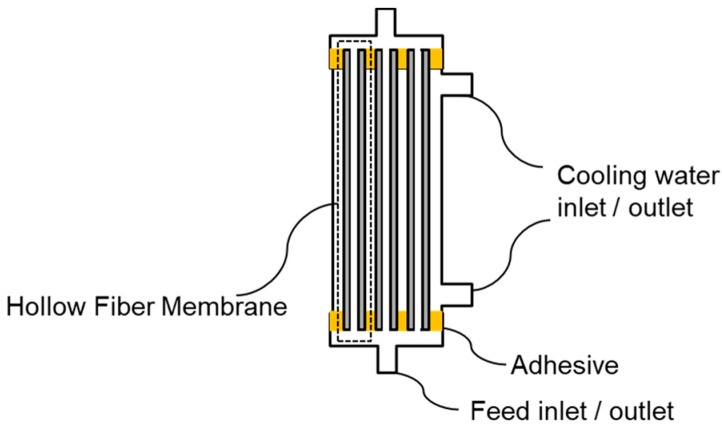
Schematic of membrane modules for DCMD.

A laboratory-scale module was constructed by inserting 70 PVDF HF membranes with a length of 11cm into a pipe and curing both ends with a urethane adhesive [18]. The total bore surface area of the membrane in the lab-scale module was 0.012 m^2^ (Figure 2).

#### 2.2.3. Hydrophobic Treatment

**Figure 3:**
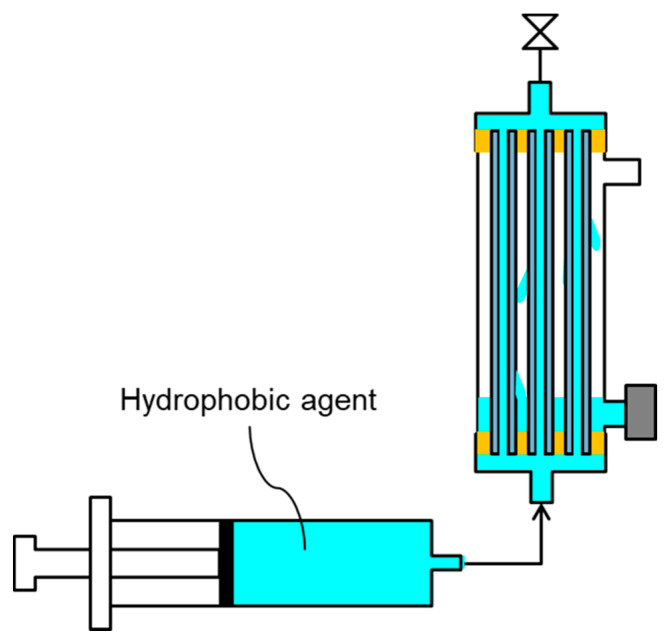
Schematic diagram of hydrophobic treatment.

One side of the feed inlet/outlet of the module was sealed, following which a hydrophobic agent was injected by a syringe into the bore side of the hollow fiber membranes from the other side of the feed inlet/outlet of the module to wet the whole membrane (Figure 3) [18]. Before injecting, the hydrophobic agent, fluoropolymer FS-392B was concentrated up to three times using an evaporator [26]. Additionally, a permeated hydrophobic agent wet the outer surface of the HF membranes. After wetting the entire membrane, the excess hydrophobic agent was removed. Using dry air flowing into the module, the membrane was then dried overnight at a temperature of ≈25 °C to obtain a hydrophobized PVDF HF membrane module. This operation hydrophobized entire HF membrane, including the bore surface, shell surface and cross section of the membrane.

### 2.3. Characterization of PVDF Membrane

#### 2.3.1. Liquid Entry Pressure (LEP) Measurement

**Figure 4:**
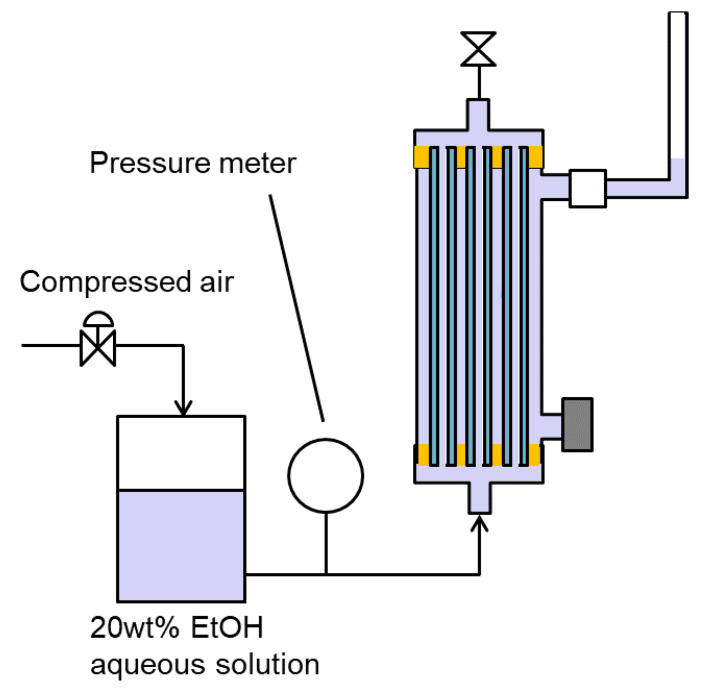
How to measure LEP of membrane.

To measure the LEP of the membrane, both the bore side and the shell sides were filled with 20 wt% ethanol aqueous solution, following which pressure was applied to the bore side (Figure 4). We used a 20 wt% ethanol aqueous solution to simulate actual operating conditions for LEP evaluation. The pressure was gradually increased while the liquid level in the tube connected to the module’s shell outlet was monitored. LEP was determined as the pressure at which the liquid level in the tube began to rise [18].

#### 2.3.2. DCMD Evaluation

**Figure 5:**
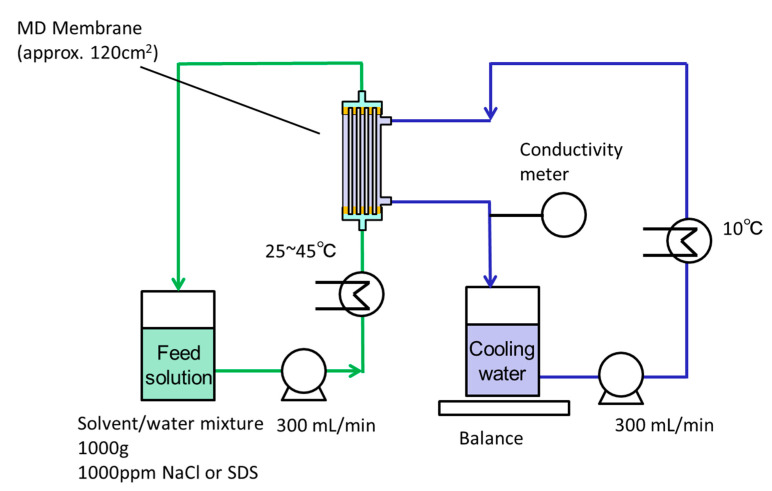
The schematic of DCMD system.

The MD performance was evaluated using the equipment shown in Figure 5 [26]. The feed organic solvent/water mixture (1000 g) was heated to the desired temperature (25–45 °C) and circulated at a flow rate of 300 mL/min to the bore side of the membrane module. The feed solution contained 1000 ppm NaCl or SDS. The temperature of the cooling water (1000 g) was lowered to <10 °C and circulated at a flow rate of 300 mL/min to the shell side of the membrane module. The higher flow rate of feed and coolant is better to acquire higher vapor flux, since the effect of heat conduction is decreased. However, an applied pressure to the inlet of the HF membrane must be increased to increase the flow rate, resulting in the increase of transmembrane pressure and the increase of risk of membrane wetting. From the balance between the merit and demerit of high flow rate, 300 mL/min in the flow rate was chosen for the feed and the coolant. The total permeate vapor flux (sum of the water and organic solvents vapor fluxes) through the membrane, $J_{p}$ (kg/m^2^·h) was given by Equation (3):

(3) $$J_{p}=\frac{W_{p}}{A\cdot t}=\frac{W_{c\;}-W_{c0}}{A\cdot t}$$

Here, $W_{p}$ (kg) is the weight of permeate, $W_{c0}$ (kg) and $W_{c}$ (kg) are the weights of cooling water before and after the operation, respectively. *A* (m^2^) is the total membrane bore surface area, and *t* (h) is the operating time.

The flux of total leaking solute, $J_{s}$ (g/m^2^·h) was given by Equation (4):

(4) $$J_{s}=\frac{1000\Delta m_{s}}{A\cdot t}=\frac{1000(W_{c\;}\cdot C_{c}-W_{c0\;}\cdot C_{c0})}{A\cdot t}$$

Here $\Delta m_{s}$ (kg) is the difference of the amount of solute contained in cooling water before and after the operation. $C_{c0}$ (wt%), and $C_{c}$ (wt%) are the solute concentrations in cooling water before and after the operation, respectively, which were obtained from the conductivity of the cooling water.

The concentration factor *F*, and the solute retention ratio in the feed, β (%) were given by Equations (5) and (6), respectively. β (%) is also confirmed from the leaking solute flux given by Equation (4).

(5) $$F=\frac{C_{f}}{C_{f0}}$$

(6) $$\beta =\frac{m_{f}}{m_{f0}}\times 100=\frac{W_{f}\;\cdot \;C_{f}}{W_{f0}\;\cdot \;C_{f0}}\times 100$$

Here, $m_{f0}$ (kg) and $m_{f}$ (kg) are the amounts of solute in the feed before and after the operation, respectively. $C_{f0}$ (wt%) and $C_{f}$ (wt%) are the solute concentrations in the feed before and after the operation, respectively, which were obtained from the conductivity of the feed. $W_{f0}$ (kg) and $W_{f}$ (kg) are the weights of the feed before and after the operation, respectively.

The permeate vapor flux of organic solvent, $J_{os}$ (kg/m^2^·h) was given by Equation (7):

(7) $$J_{os}=\frac{W_{\mathrm{os}}}{A\cdot t}=\frac{\left(W_{c\;}\cdot C_{os}-W_{c0\;}\cdot C_{os0}\right)}{A\cdot t}$$

Here, $W_{os}$ (kg) is the weight of permeated organic solvent through membrane. $C_{os0}$ (wt%) and $C_{os}$ (wt%) are the organic solvent concentrations of cooling water before and after the operation, respectively. The organic solvent concentration was measured using the refractive index meter PAL-RI (ATAGO Co., Ltd., Tokyo, Japan). The accuracy of organic solvent concentration is about ±0.5wt% for ethanol and ± 0.6wt% for acetonitrile due to the measurement accuracy of PAL-RI.

The concentration of organic solvent in vapor $C_{osv}$ (%) was given by Equation (8):

(8) $$C_{osv}=\frac{J_{os}}{J_{p}}\times 100$$

## 3. Results and Discussion

### 3.1. Membrane Morphology and Membrane Properties

**Figure 6:**
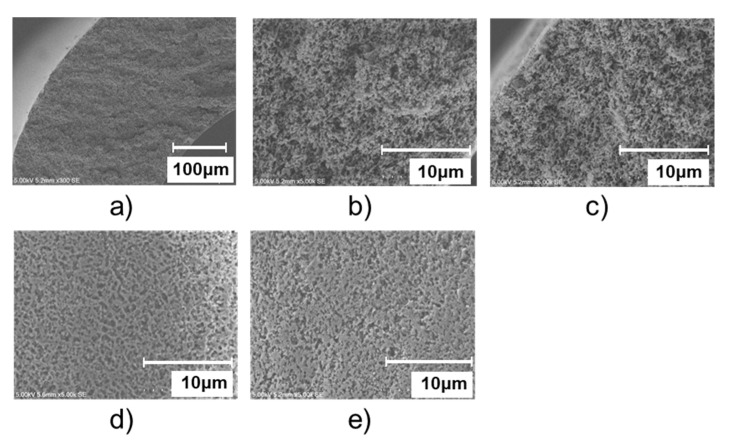
Membrane morphology. (**a**) Cross section, (**b**) Near the bore side of the cross section, (**c**) Near the shell side of the cross section, (**d**) Bore surface, (**e**) Shell surface.

Following a hydrophobic treatment, the PVDF HF membrane, described in a previous paper, was used in this study [18]. The morphology of the membrane is depicted in Figure 6, and its properties are listed in Table 1. As illustrated in Figure 6a–c, the membrane had a highly porous and uniform sponge-like structure throughout its cross-section. Additionally, the bore surface porosity was observed to be higher than that of the shell surface (Figure 6d,e). Furthermore, due to the hydrophobic treatment, the water contact angle (132°) was higher than that of original PVDF membranes (103°) (Table 1). As a result, the LEP of this membrane was quite high even when used with a 20 wt% ethanol aqueous solution (0.24 MPa), for which the original PVDF membrane was easily wetted.

### 3.2. MD Performance

#### 3.2.1. Effect of Operating Temperature on MD Performance

DCMD operations were carried out using the DCMD system shown in Figure 5. The effect of feed temperature on DCMD performance was investigated at first, maintaining a coolant temperature of ≈10 °C. The experiment used a 1000 ppm NaCl aqueous solution as feed, with NaCl serving as both a model valuable solute and an indicator of membrane wetting. Since NaCl is difficult to vaporize within the temperature range of MD operation, it is suitable for the valuable model solutes. Furthermore, NaCl is also a suitable indicator of membrane wetting because the size of the NaCl molecule is much smaller than the pore size of MD membrane (≈0.1 μm) [18]. Thus, when the MD is not wet, NaCl does not permeate through the membrane. However, it easily permeates when the membrane is wet. Additionally, NaCl permeation can be detected easily by measuring the conductivity of the coolant.

The results of the DCMD test with various feed temperatures are shown in Table 2. In all conditions, the operation time was maintained at 2 h. When the feed-in temperature was 25.1 °C, the water vapor flux through the membrane was 0.8 kg/m^2^·h. It further increased to 3.1 kg/m^2^·h as the feed-in temperature increased to 45.4 °C. Thus, the vapor flux increased with the feed temperature when the temperature of the coolant was kept constant. This is due to the increase in the vapor pressure difference between the feed and the cooling water, which is the driving force for vapor permeation across the membrane.

Additionally, the leaking salt flux was less than 0.01 g/m^2^·h and the solute retention ratio in the feed was over 99.9%, in all conditions. This demonstrates that solutes, as small as Na^+^ and Cl^−^ ions, can be maintained at a concentration of ≈100% in the feed.

#### 3.2.2. MD Performance with Organic Solvent/Water Mixture at Various Temperatures

To confirm whether DCMD was capable of operating in an aqueous solution containing an organic solvent, ethanol and acetonitrile were used as the model organic solvents and the impact of the feed temperature on the membrane performance was investigated, with a coolant temperature of 10 °C. In all conditions, the feed contained 15 wt% ethanol or acetonitrile, and 1000 ppm NaCl was also used as the model valuable solute. The DCMD operation was performed for 2 h at three different feed temperatures, 25, 35 and 45 °C.

As shown in Table 3, after two hours of operation, the leaking solute flux was less than 0.01 g/m^2^∙h and the solute retention ratio was greater than 99.9% in all conditions. These performances were high enough to apply for solute recovery and are comparable to the conventional OSN processes [7,8,9]. The results, thereby indicate that, despite the presence of an organic solvent in the feed, the solute is recovered sufficiently. This strongly suggests that the hydrophobic membrane is easy to maintain high a LEP, and that the DCMD mode can avoid excessive transmembrane pressure.

**Figure 7:**
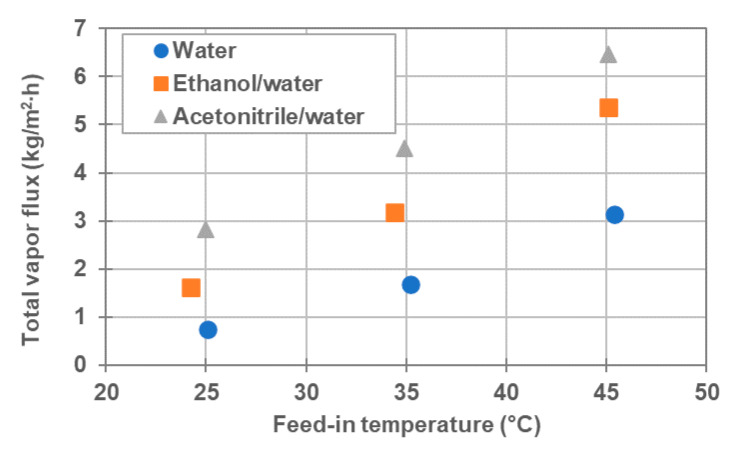
Total vapor flux for three kind of feeds, water (Table 2), ethanol/water and acetonitrile/water (Table 3), as a function of feed-in temperature.

Furthermore, the total vapor flux increased as the feed-in temperature increased, even in the presence of an organic solvent. Figure 7 shows the total vapor flux for three kinds of feeds as a function of feed-in temperature. As illustrated, the total vapor flux was observed to increase due to the inclusion of the organic solvent. Additionally, it was found that the total vapor flux for the mixture of acetonitrile/water mixture is slightly higher than that for ethanol/water.

These phenomena are qualitatively explained by Raoult’s law and Dalton’s law. In the case of an ideal solution, according to the Raoult’s law, the partial vapor pressure of component *i* in the mixture, *P_i_* (kPa) is given by Equation (9). The total vapor pressure, $P_{total}\;\left(\mathrm{kPa}\right)$ is given by Equation (10) according to Dalton’s law.

(9) $$P_{i}=P_{i}^{0}\chi_{i}$$

(10) $$P_{total}=\sum P_{i}=\sum P_{i}^{0}\chi_{i}$$

where $P_{i}^{0}$ (kPa) indicates the vapor pressure of pure liquid and $\chi_{i}$ the molar fraction of component *i*. In the case of non-ideal solution such as ethanol/water mixture and acetonitrile/water mixture, it is necessary to consider the activity coefficient γ, and Equation (9) is rewritten by Equation (11) [27].

(11) $$P_{i}=\gamma_{i}P_{i}^{0}\chi_{i}$$

where $\gamma_{i}$ indicates the activity coefficient of component *i*. Activity coefficients can be calculate by Wilson equation [28]. In the case of the binary mixture of components *i* and *j*, the activity coefficient of those components $\gamma_{i}$ and $\gamma_{j}$ are given by Equations (12) and (13), respectively.

(12) $$\ln\gamma_{i}=-\ln\left(\chi_{i}+\Lambda_{ij}\chi_{j}\right)+\chi_{j}\left(\frac{\Lambda_{ij}}{\chi_{i}+\Lambda_{ij}\chi_{j}}-\frac{\Lambda_{ji}}{\;\Lambda_{ji}\chi_{i}+\chi_{j}}\right)$$

(13) $$\ln\gamma_{j}=-\ln\left(\Lambda_{ji}\chi_{i}+\chi_{j}\right)-\chi_{i}\left(\frac{\Lambda_{ij}}{\chi_{i}+\Lambda_{ij}\chi_{j}}-\frac{\Lambda_{ji}}{\;\Lambda_{ji}\chi_{i}+\chi_{j}}\right)$$

where $\Lambda_{ij}$ and $\Lambda_{ji}$ indicate the Wilson parameters. Those parameters are given by Equation (14).

(14) $$\Lambda_{ij}=\;exp\left(a_{ij}+\frac{b_{ij}}{T}\right)$$

where $a_{ij}$ and $b_{ij}$ indicate the parameter coefficients, which are determined by the combination of the components *i* and *j*. *T* indicates absolute temperature [29]. Table 4 shows $a_{ij}$, $a_{ji}$, $b_{ij}$ and $b_{ji}$ of ethanol/water mixture and acetonitrile/water mixture, which can be obtained from Aspen plus^®^ [30].

**Figure 8:**
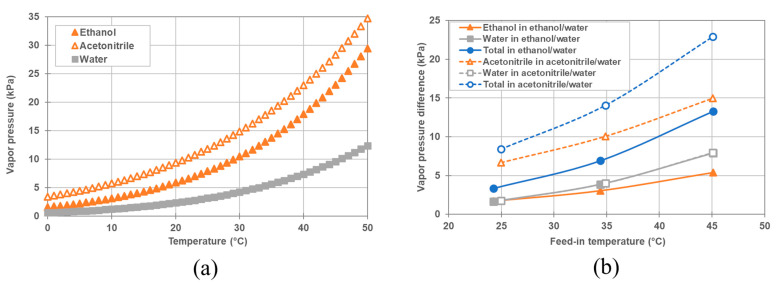
(**a**) Vapor pressure of acetonitrile, ethanol, water as a function of temperature. (**b**) Vapor pressure differences for ethanol/water and acetonitrile/water systems between feed side and permeate side in the beginning of operation under the condition shown in Table 3. Open and closed symbols show the vapor pressure difference of acetonitrile/water mixture, and ethanol/water mixture, respectively. Triangles shows the partial vapor pressure difference of organic solvent, squares the partial vapor pressure difference of water and circles the total vapor pressure difference.

Figure 8a shows the vapor pressure, *P_i_*^0^, of pure ethanol, acetonitrile and water as a function of temperature, calculated using the Antoine equation, Equation (15) [16].

(15) $$log\left(\frac{P_{i}^{0}}{100}\right)=\;\left(A_{i}-\frac{B_{i}}{T+C_{i}}\right)$$

where $A_{i}$, $B_{i}$ and $C_{i}$ are constants of Antoine’s equation, 100 is a factor to convert “kPa” to “bar”, since *P_i_* is in “kPa”, and the pressure calculated with parameter shown in Table 5 is in “bar”. Table 5 shows $A_{i}$, $B_{i}$ and $C_{i}$ of ethanol, acetonitrile and water, which can be obtained from the website of National Institute of Standards and Technology (NIST) [31].

As observed from Figure 8a, the order of vapor pressure of pure liquid is acetonitrile > ethanol > water across all temperature ranges.

Furthermore, the activity coefficient and vapor pressure of organic solvent and water were calculated using the feed-in temperature, coolant-in temperature and molar fraction as operating conditions and are shown in Table 6.

Figure 8b illustrates the vapor pressure differences between the feed and permeate sides of ethanol/water and acetonitrile/water systems, as a function of feed-in temperature at startup under the experimental conditions specified in Table 3. In Figure 8b, the vapor pressure is calculated using the feed-in temperature and the coolant-in temperature. The molar fractions of organic solvents in the feed were set to 0.072 and 0.065 for acetonitrile and ethanol, respectively, based on a 15 wt% aqueous solution. As the solvent concentration is zero at startup, the molar fractions of the organic solvents were put as zero on the permeate side. To simplify the calculation, a small contribution of the vapor pressure drop by NaCl in the feed was ignored. According to Figure 8a,b, the total vapor pressure difference between the feed and permeate sides of the organic solvent/water mixture was greater than that between the feed and permeate sides of pure water, at the corresponding temperature under the experimental conditions listed in Table 3. In addition, the total vapor pressure difference of acetonitrile/water mixture was greater than that of ethanol/water. As a result, it is qualitatively understood that the reason for the increase in total vapor flux, caused by the addition of the organic solvent, is that the total vapor pressure difference increases. In addition, the reason for the total vapor flux for the acetonitrile/water mixture being slightly larger than that for the ethanol/water mixture was attributed to the difference in the total vapor pressure difference. As shown in Table 6 and Figure 8b, the partial vapor pressure difference for acetonitrile was greater than that of ethanol. This is ascribed to the larger fraction of acetonitrile (0.072) in the mixture in comparison to ethanol (0.065), as well as, to the larger activity coefficient of acetonitrile. Thus, as shown in Table 3, the vapor flux of acetonitrile was higher than that of ethanol at similar feed-in temperatures. Moreover, the ratio of organic solvent vapor flux to the total vapor flux was comparable to or greater than that of water vapor flux, even though the molar fraction of organic solvent was lower than that of water. When DCMD was operated at around 25 °C of feed-in temperature, the ratio of the acetonitrile and ethanol vapor fluxes to the total vapor flux exceeded 60%. This is because ethanol and acetonitrile have a much higher activity coefficient than water in an organic solvent/water mixture, as a result of which the partial vapor pressure of organic solvents becomes comparable or higher than that of water.

#### 3.2.3. MD Performance with Various Compositions of Organic Solvent

**Figure 9:**
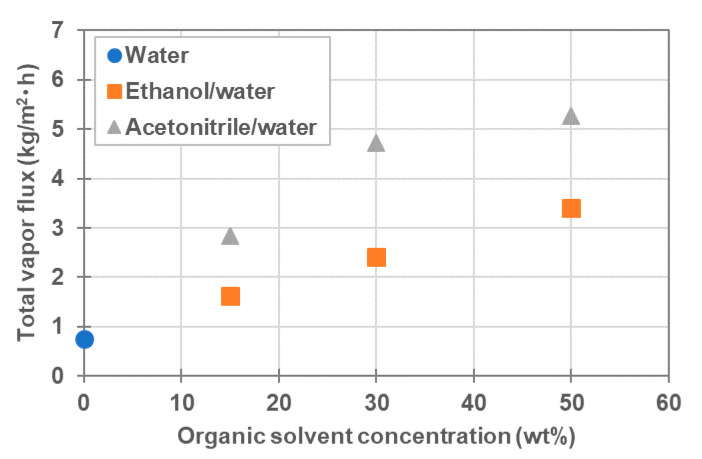
The total vapor flux vs. organic solvent composition of the feed. The data for water was from Table 2.

The effect of the concentration of organic solvents in the feed was also investigated. Herein, the organic solvent/water mixture containing 1000 ppm NaCl was used as the feed, and the feed-in temperature and the coolant-in temperature were set to ≈25 °C and ≈10 °C, respectively. The concentrations of ethanol and acetonitrile in the mixture were varied from 15–50 wt%. The experimental conditions and results are listed in Table 7. The total vapor flux is plotted in Figure 9 as a function of the concentration of organic solvents, including pure water. As observed, the total vapor flux of organic solvent/water mixture was higher than that of pure water and increased as the organic solvent fraction increased. Additionally, as shown in Table 7, the organic solvent flux increased as the organic solvent fraction increased. Moreover, the total vapor flux and the organic solvent flux for the acetonitrile/water mixture were higher than for the ethanol/water mixture.

**Figure 10:**
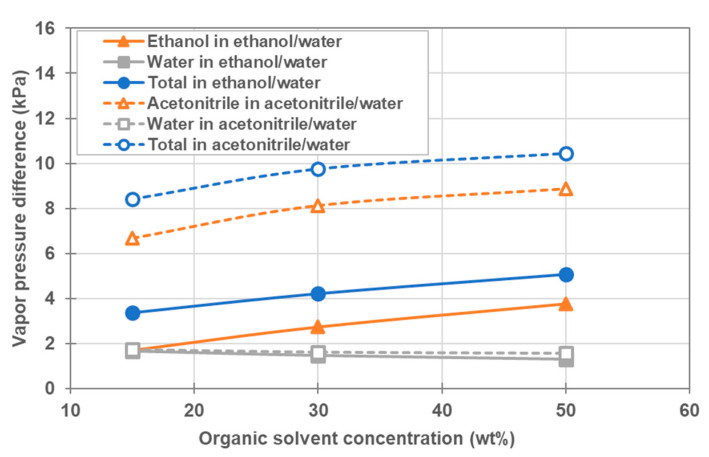
Vapor pressure differences between feed side and permeate side of ethanol/water and acetonitrile/water systems in the beginning of operation under the condition shown in Table 7. Open and closed symbols show the vapor pressure difference of acetonitrile/water mixture, and ethanol/water mixture, respectively. Triangles show the partial vapor pressure difference of organic solvent, squares the partial vapor pressure difference of water and circles the total vapor pressure difference.

These phenomena are qualitatively explained as follows: As shown in Equation (11), the partial vapor pressure increases with the molar fraction and the activity coefficient. Table 8 shows the activity coefficient, and vapor pressure of organic solvent and water in the feed and the permeate, which were calculated from the operating conditions of feed-in temperature, coolant-in temperature and molar fraction shown in Table 7. As shown in Table 8, the molar fraction of acetonitrile is higher than that of ethanol in the mixture with the same wt%, and the activity coefficient of acetonitrile is higher than that of ethanol. In addition, as shown in Figure 8a, the vapor pressure of pure acetonitrile is higher than that of pure ethanol at the same temperature. Thus, the partial vapor pressure of acetonitrile is higher than that of ethanol in the organic solvent/water mixture with the same wt% at the same temperature. To clearly show this situation, Figure 10 shows the vapor pressure difference at the startup of operation, under each condition. It is obvious from Figure 10 that the total vapor pressure difference and the partial vapor pressure difference of the organic solvent simultaneously increased with the increase in its molar fraction. In addition, the partial vapor pressure differences of acetonitrile and of ethanol are higher than that of water, and the partial vapor pressure difference of acetonitrile is higher than that of ethanol. Consequently, the total vapor flux and the solvent flux simultaneously increased with the increase of the molar fraction of the organic solvent. In addition, the total vapor flux and the solvent flux for the acetonitrile/water mixture were higher than those of the ethanol/water mixture, since the total vapor pressure difference and the partial vapor pressure difference of the acetonitrile/water organic solvent mixture were higher than those of the ethanol/water mixture.

Surprisingly, as demonstrated in Table 7, even when the feed contains 50 wt% ethanol or acetonitrile, the retention ratio of the solute was over 99.9%. This demonstrates that DCMD can be used to recover valuable solutes without losing any solute, even under such harsh conditions for the MD membrane.

#### 3.2.4. Effect of Surfactant on MD Performance

Polysaccharides [32], phospholipids [33] and peptides [34] comprise valuable surface-active solutes. Surfactants are notoriously difficult to recover using MD because they significantly reduce the surface tension of the aqueous solution, increasing the risk of membrane wetting during MD operation.

The effect of the surfactant in the feed on MD performance was investigated in this section to confirm the possibility of surface-active solute recovery via MD. The DCMD operation was performed for 2 h with the feed containing 1000 ppm SDS as the model valuable surface-active solute along with 15 wt% acetonitrile, at feed and coolant temperatures of ≈25 °C and ≈10 °C, respectively. The experimental condition and result are listed in Table 9. No SDS leakage was observed after two hours of operation, as indicated by the change in the cooling water conductivity. This result, therefore, indicates that DCMD with this membrane will be used to effectively recover surface-active solutes.

## 4. Conclusions

Recently, the demand for the recovery of valuable solutes from organic solvents/water mixtures has increased in various fields. Furthermore, due to the abundance of heat-sensitive valuable solutes, the demand for non-heated concentration technologies has increased. The current study investigated DCMD operation using hydrophobized PVDF HF membranes, to confirm a possibility of recovering heat-sensitive valuable solutes from organic solvents/water mixtures via MD as a non-heated process. At first, the possibility of DCMD operation at low feed temperature was evaluated using 1000 ppm NaCl aqueous solution as the feed, and it was confirmed DCMD could achieve 0.8 kg/m^2^·h of vapor flux even at feed and coolant temperatures of 25 °C and 10 °C, respectively. Furthermore, the NaCl retention ratio was observed to be >99.9%, indicating that it was possible to operate DCMD at low feed temperature. Subsequently, the recovery of solutes from organic solvent/water mixtures was evaluated using ethanol/water and acetonitrile/water mixtures containing 1000 ppm NaCl. As a result, it was confirmed that DCMD could be applied for the recovery of solutes from organic solvent/water mixtures without causing membrane wetting or solute leakage. The effect of feed temperature (25, 35, 45 °C) and concentration of organic solvents (15, 30, 50 wt%) were also investigated using ethanol/water and acetonitrile/water mixtures containing 1000 ppm NaCl. The total vapor flux, as well as the partial vapor flux of organic solvents simultaneously increased with the temperature and concentration of the organic solvents. These phenomena were qualitatively explained by changes in partial vapor pressure of organic solvent in the organic solvent/water mixtures. Additionally, there was no solute leakage under any of the conditions. Furthermore, investigations using SDS as a model valuable surface-active solute also demonstrated the possibility of recovering surface-active solutes from organic solvent/water mixtures via DCMD. These findings, therefore, indicate that DCMD with a hydrophobic hollow fiber membrane will be applied for the recovery of valuable solutes from organic solvent/water mixtures as a non-heated process even under harsh condition where surface active solutes are included in the feed.

## Figures and Tables

**Table 1:** Membrane properties.

Membrane	OD ^1^	ID ^2^	Thickness	Contact Angle ^3^	LEP ^4^
	[mm]	[mm]	[mm]	[°]	[MPa]
Before hydrophobic treatment	1.25	0.68	0.28	103	N/A
After hydrophobic treatment	1.25	0.68	0.28	132	0.24

^1^ Outer diameter, ^2^ Inner diameter, ^3^ water, ^4^ for 20 wt% ethanol aqueous solution.

**Table 2:** Results of DCMD operation with 1000 ppm NaCl aqueous solution as feed at various temperature.

Temperature				Operating Time	Water Vapor Flux	Leaking Solute (NaCl) Flux	Concentration Factor	Solute (NaCl) Retention Ratio in Feed
Feed-In ^1^	Feed-Out ^2^	Coolant-In ^3^	Coolant-Out ^4^					
[°C]	[°C]	[°C]	[°C]	[h]	[kg/m^2^∙h]	[g/m^2^∙h]	-	%
25.1	23.4	11.5	12.9	2.0	0.8	<0.01	1.02	>99.9%
35.2	32.3	11.6	13.8	2.0	1.7	<0.01	1.04	>99.9%
45.4	40.4	12.8	17.1	2.0	3.1	<0.01	1.08	>99.9%

^1^ Temperature of the feed at module inlet. ^2^ Temperature of the feed at module outlet. ^3^ Temperature of the cooling water at module inlet. ^4^ Temperature of the cooling water at module outlet.

**Table 3:** Results of DCMD operation at various temperature with the feed which contains 1000 ppm NaCl and 15 wt% of ethanol or acetonitrile.

**Organic Solvent**	**Temperature**				**Operating Time**	**Vapor Flux**		
	**Feed-In ^1^**	**Feed-Out ^2^**	**Coolant-In ^3^**	**Coolant-Out ^4^**		**Total**	**Water**	**Organic Solvent**
	**[°C]**	**[°C]**	**[°C]**	**[°C]**	**[h]**	**[kg/m^2^∙h]**	**[kg/m^2^∙h]**	**[kg/m^2^∙h]**
Ethanol	24.3	22.1	9.8	11.3	2.0	1.6	0.6	1.1
	34.4	30.7	10.7	13.9	2.0	3.2	1.6	1.6
	45.1	39.0	10.5	15.7	2.0	5.4	3.0	2.4
Acetonitrile	25.0	22.8	10.8	12.4	2.0	2.8	0.6	2.2
	34.9	29.8	11.4	15.1	2.0	4.5	1.6	2.9
	45.1	38.9	10.4	15.5	2.0	6.5	3.1	3.3
**Organic solvent**	**Organic solvent conc. in feed**				**Leaking solute (NaCl) flux**		**Concentration factor**	**Solute (NaCl) retention ratio in Feed**
	**Before operation**		**After operation**					
	**[wt%]**		**[wt%]**		**[g/m^2^∙h]**		**-**	**%**
Ethanol	15.0		12.9		<0.01		1.04	>99.9%
	15.0		12.2		<0.01		1.08	>99.9%
	15.0		10.7		<0.01		1.15	>99.9%
Acetonitrile	15.0		10.4		<0.01		1.07	>99.9%
	15.0		9.0		<0.01		1.12	>99.9%
	15.0		8.3		<0.01		1.18	>99.9%

^1^ Temperature of the feed at module inlet. ^2^ Temperature of the feed at module outlet. ^3^ Temperature of the cooling water at module inlet. ^4^ Temperature of the cooling water at module outlet.

**Table 4:** The parameter coefficients for calculation of Wilson parameters of ethanol/water mixture and acetonitrile/water mixture.

Component *i*	Component *j*	$a_{ij}$	$a_{ji}$	$b_{ij}$	$b_{ji}$
Ethanol	Water	−2.5035	−0.0503	346.151	−69.6372
Acetonitrile	Water	−0.8487	1.0158	−386.606	−707.346

**Table 5:** The constants of Antoine’s equation of ethanol, acetonitrile and water. By using these numbers, the unit of obtained vapor pressure is bar.

Component	*A*	*B*	*C*
Ethanol	5.93296	2345.829	43.815
Acetonitrile	5.37229	1670.409	−40.191
Water	5.40221	1838.675	−31.737

**Table 6:** The activity coefficient, vapor pressure of organic solvent and water, calculated from the operating condition of feed-in temperature and coolant-in temperature of the module, and mol-fraction.

**Organic Solvent**	**Feed-In ^1^**								
	**Temperature**	**Organic Solvent Conc.**	**Molar Fraction**		**Activity Coefficient**		**Vapor Pressure**		
			**Organic Solvent**	**Water**	**Organic Solvent**	**Water**	**Organic Solvent**	**Water**	**Total**
	**[°C]**	**[wt%]**	**-**	**-**	**-**	**-**	**[kPa]**	**[kPa]**	**[kPa]**
Ethanol	24.3	15.0	0.065	0.935	3.5	1.0	1.7	2.9	4.6
	34.4	15.0	0.065	0.935	3.6	1.0	3.1	5.2	8.2
	45.1	15.0	0.065	0.935	3.6	1.0	5.4	9.1	14.6
Acetonitrile	25.0	15.0	0.072	0.928	7.9	1.0	6.7	3.0	9.7
	34.9	15.0	0.072	0.928	7.6	1.0	10.1	5.3	15.4
	45.1	15.0	0.072	0.928	7.3	1.0	14.9	9.2	24.1
**Organic solvent**	**Coolant-in ^2^**							**Vapor pressure difference**	
	**Temperature**	**Molar fraction**		**Activity coefficient**		**Vapor pressure**			
		**Organic solvent**	**Water**	**Organic solvent**	**Water**	**Organic solvent**	**Water**	**Organic solvent**	**Water**
	**[°C]**	**-**	**-**	**-**	**-**	**[kPa]**	**[kPa]**	**[kPa]**	**[kPa]**
Ethanol	9.8	0.000	1.000	-	1.0	-	1.2	1.7	1.7
	10.7	0.000	1.000	-	1.0	-	1.3	3.1	3.9
	10.5	0.000	1.000	-	1.0	-	1.3	5.4	7.9
Acetonitrile	10.8	0.000	1.000	-	1.0	-	1.3	6.7	1.7
	11.4	0.000	1.000	-	1.0	-	1.4	10.1	4.0
	10.4	0.000	1.000	-	1.0	-	1.3	14.9	7.9

^1^ The property of the feed at module inlet. ^2^ The property of the cooling water at module inlet.

**Table 7:** Results of DCMD operation at about 25 °C with the feed which contains 1000 ppm NaCl and various concentration of ethanol or acetonitrile.

**Organic Solvent**	**Temperature**				**Operating Time**	**Vapor Flux**		
	**Feed-In ^1^**	**Feed-Out ^2^**	**Coolant-In ^3^**	**Coolant-Out ^4^**		**Total**	**Water**	**Organic Solvent**
	**[°C]**	**[°C]**	**[°C]**	**[°C]**	**[h]**	**[kg/m^2^∙h]**	**[kg/m^2^∙h]**	**[kg/m^2^∙h]**
Ethanol	24.3	22.1	9.8	11.3	2.0	1.6	0.6	1.1
	24.0	21.9	9.8	11.4	2.0	2.4	0.5	1.9
	24.2	21.9	9.8	11.3	2.0	3.4	0.8	2.6
Acetonitrile	25.0	22.8	10.8	12.4	2.0	2.8	0.6	2.2
	24.3	22.2	9.9	12.0	2.0	4.7	1.2	3.6
	24.3	22.1	9.7	11.7	2.0	5.3	1.0	4.2
**Organic solvent**	**Organic solvent conc. in feed**				**Leaking solute (NaCl) flux**	**Concentration factor**	**Solute (NaCl) retention ratio in Feed**	
	**Before operation**		**After operation**					
	**[wt%]**		**[wt%]**		**[g/m^2^∙h]**	**-**	**%**	
Ethanol	15.0		12.9		<0.01	1.04	>99.9%	
	30.0		26.9		<0.01	1.06	>99.9%	
	50.0		47.6		<0.01	1.09	>99.9%	
Acetonitrile	15.0		10.4		<0.01	1.07	>99.9%	
	30.0		24.2		<0.01	1.13	>99.9%	
	50.0		45.6		<0.01	1.14	>99.9%	

^1^ Temperature of the feed at module inlet. ^2^ Temperature of the feed at module outlet. ^3^ Temperature of the cooling water at module inlet. ^4^ Temperature of the cooling water at module outlet.

**Table 8:** The activity coefficient, vapor pressure of organic solvent and water, calculated from the operating conditions of feed-in temperature and mol-fraction.

**Organic Solvent**	**Feed-In ^1^**								
	**Temperature**	**Organic Solvent Conc.**	**Molar Fraction**		**Activity Coefficient**		**Vapor Pressure**		
			**Organic Solvent**	**Water**	**Organic Solvent**	**Water**	**Organic Solvent**	**Water**	**Total**
	**[°C]**	**[wt%]**	**-**	**-**	**-**	**-**	**[kPa]**	**[kPa]**	**[kPa]**
Ethanol	24.3	15.0	0.065	0.935	3.5	1.0	1.7	2.9	4.6
	24.0	30.0	0.143	0.856	2.6	1.0	2.7	2.7	5.4
	24.2	50.0	0.281	0.719	1.8	1.2	3.8	2.5	6.3
Acetonitrile	25.0	15.0	0.072	0.928	7.9	1.0	6.7	3.0	9.7
	24.3	30.0	0.158	0.841	4.5	1.1	8.1	2.8	11.0
	24.3	50.0	0.305	0.695	2.5	1.3	8.9	2.8	11.6
**Organic solvent**	**Coolant-in ^2^**							**Vapor pressure difference**	
	**Temperature**	**Molar fraction**		**Activity coefficient**		**Vapor pressure**			
		**Organic solvent**	**Water**	**Organic solvent**	**Water**	**Organic solvent**	**Water**	**Organic solvent**	**Water**
	**[°C]**	**-**	**-**	**-**	**-**	**[kPa]**	**[kPa]**	**[kPa]**	**[kPa]**
Ethanol	9.8	0.000	1.000	-	1.0	-	1.2	1.7	1.7
	9.8	0.000	1.000	-	1.0	-	1.2	2.7	1.5
	9.8	0.000	1.000	-	1.0	-	1.2	3.8	1.3
Acetonitrile	10.8	0.000	1.000	-	1.0	-	1.3	6.7	1.7
	9.9	0.000	1.000	-	1.0	-	1.2	8.1	1.6
	9.7	0.000	1.000	-	1.0	-	1.2	8.9	1.6

^1^ The property of the feed at module inlet. ^2^ The property of the cooling water at module inlet.

**Table 9:** Results of DCMD operation at 25 °C with the feed which contains 1000 ppm SDS and 15wt% of acetonitrile.

**Organic Solvent**	**Temperature**				**Operating Time**	**Vapor Flux**		
	**Feed-In ^1^**	**Feed-Out ^2^**	**Coolant-In ^3^**	**Coolant-Out ^4^**		**Total**	**Water**	**Organic Solvent**
	**[°C]**	**[°C]**	**[°C]**	**[°C]**	**[h]**	**[kg/m^2^∙h]**	**[kg/m^2^∙h]**	**[kg/m^2^∙h]**
Acetonitrile	24.5	22.1	10.1	12.0	2.0	3.2	0.8	2.4
**Organic solvent**	**Organic solvent conc. in feed**				**Leaking solute (SDS) flux**	**Concentration factor**		**Solute (SDS) retention ratio in Feed**
	**Before operation**		**After operation**					
	**[wt%]**		**[wt%]**		**[g/m^2^∙h]**	**-**		**%**
Acetonitrile	15.0		10.1		<0.01	1.08		>99.9%

^1^ Temperature of the feed at module inlet. ^2^ Temperature of the feed at module outlet. ^3^ Temperature of the cooling water at module inlet. ^4^ Temperature of the cooling water at module outlet.

## Data Availability

Not applicable.

## Nomenclature

Symbols	Description	Units
*A*	Total membrane bore surface area	m^2^
*B*	Geometric factor determined by pore structure	–
$C_{c0}$	Solute concentration in cooling water before operation	wt%
$C_{c}$	Solute concentration in cooling water after operation	wt%
$C_{os0}$	Organic solvent concentration of cooling water before operation	wt%
$C_{f0}$	Solute concentration in the feed before operation	wt%
$C_{f}$	Solute concentration in the feed after operation	wt%
$C_{os}$	Organic solvent concentration of cooling water after operation	wt%
$C_{osv}$	Concentration of organic solvent in vapor	%
$A_{i}$, $B_{i}$, $C_{i}$	Constants of Antoine’s equation of component *i*	–
$a_{ij}$, $a_{ji}$, $b_{ij}$, $b_{ji}$	parameter coefficients for calculate Wilson parameter	–
*F*	Concentration factor	fold
$J_{os}$	Permeate vapor flux of organic solvent through the membrane	kg/m^2^·h
$J_{p}$	Total permeate vapor flux (water and organic solvents) through the membrane	kg/m^2^·h
$J_{s}$	Flux of total leaking solute through the membrane	g/m^2^·h
LEP	Liquid Entry Pressure	MPa
$m_{f0}$	Amount of solute in the feed before operation	kg
$m_{f}$	Amount of solute in the feed after operation	kg
$\Delta m_{s}$	Difference of the amount of solute contained in cooling water before and after operation	kg
$P_{\mathrm{feed}}$	Saturated vapor pressure of the feed side	kPa
$P_{\mathrm{permeate}}$	Saturated vapor pressure of the permeation side	kPa
$P_{i}^{0}$	Vapor pressure of pure liquid of component *i*	kPa
$P_{i}$	Partial vapor pressure of component *i*	kPa
$r_{max}$	Maximum pore radius of the membrane	μm
T	Absolute temperature	K
*t*	Operating time	hour
$W_{c}$	Weight of cooling water after operation,	kg
$W_{c0}$	Weight of cooling water before operation	kg
$W_{f0}$	Weight of the feed before operation	kg
$W_{f}$	Weight of the feed after operation	kg
$W_{p}$	Weight of permeate	kg
$W_{os}$	Weight of permeated organic solvent through membrane	kg
*α*	the vapor permeation coefficient	kg/m^2^·h·kPa
β	solute retention ratio in the feed	%
$\gamma_{i}$	activity coefficient of component *i*	–
$\sigma_{L}$	surface tension of liquid	–
θ	contact angle	°
$\Lambda_{ij}$, $\Lambda_{ji}$	Wilson parameters of the binary mixture of components *i* and *j*	–
$\chi_{i}$	molar fraction of component *i*	–
